# A Flexible Temperature Sensor Based on Reduced Graphene Oxide for Robot Skin Used in Internet of Things

**DOI:** 10.3390/s18051400

**Published:** 2018-05-02

**Authors:** Guanyu Liu, Qiulin Tan, Hairong Kou, Lei Zhang, Jinqi Wang, Wen Lv, Helei Dong, Jijun Xiong

**Affiliations:** 1 Key Laboratory of Instrumentation Science & Dynamic Measurement, Ministry of Education, North University of China, Taiyuan 030051, China; lgyydd@163.com (G.L.); 18434360348@163.com (H.K.); 18734136023@163.com (L.Z.); wjqydd@163.com (J.W.); lukelike2017@163.com (W.L.); donghelei@nuc.edu.cn (H.D.); xiongjijun@nuc.edu.cn (J.X.); 2 Science and Technology on Electronic Test and Measurement Laboratory, North University of China, Taiyuan 030051, China

**Keywords:** flexible sensor, temperature sensor, reduced graphene oxide, robot skin, IoT

## Abstract

Flexible electronics, which can be distributed on any surface we need, are highly demanded in the development of Internet of Things (IoT), robot technology and electronic skins. Temperature is a fundamental physical parameter, and it is an important indicator in many applications. Therefore, a flexible temperature sensor is required. Here, we report a simple method to fabricate three lightweight, low-cost and flexible temperature sensors, whose sensitive materials are reduced graphene oxide (r-GO), single-walled carbon nanotubes (SWCNTs) and multi-wall carbon nanotubes (MWCNTs). By comparing linearity, sensitive and repeatability, we found that the r-GO temperature sensor had the most balanced performance. Furthermore, the r-GO temperature sensor showed good mechanical properties and it could be bent in different angles with negligible resistance change. In addition, the performance of the r-GO temperature sensor remained stable under different kinds of pressure and was unaffected by surrounding environments, like humidity or other gases, because of the insulating layer on its sensitive layer. The easy-fabricated process and economy, together with the remarkable performance of the r-GO temperature sensor, suggest that it is suitable for use as a robot skin or used in the environment of IoT.

## 1. Introduction

With the continuous development of the Internet of Things (IoT) and information technology, the need for miniature sensors that can be integrated into all environments is attracting more and more attention [1,2]. Temperature, a fundamental physical parameter, plays an important role in all works of life, including the monitoring of health conditions [3,4], artificially electronic skins [5] and robot technology, as well as IOT [6]. Most temperature sensors are based on the use of certain physical changes to detect the temperature. One of the most widely utilized type of detectors is the resistive temperature detector, which has a fast response, high accuracy and good stability [7,8]. Furthermore, the use of thermal sensors [9], infrared temperature sensors [10,11] and mercury thermometers [12,13] are widespread. In addition, the main materials for these sensors are metals, metal oxides, ceramics, etc. However, they are limited by some of their own characteristics, including their inflexibility, bulkiness and fragility as well as the difficult of attaching them to the curvilinear surfaces of tested objects. Carbon-based temperature sensitive materials, including carbon black [14], graphene [15], carbon fiber, and carbon nanotubes [16] are attracting more and more attention because of their excellent mechanical and electrical properties [17]. The flexibility of sensors is essential so that they can adapt to any surface for data monitoring. The traditional way to achieve a flexible sensor, it to use a variable structure. Another method is to fix the temperature-sensitive unit to a flexible substrate, such as polydimethylsiloxane (PDMS), polyethylene terephthalate (PET), papers, textile or polyimide (PI). Robots will be widely used in the IoT and other environments in the future. Humans have great expectations for robots and hope they can help people do things like collection, transmission, and processing of information that humans cannot do in harsh environments [18]. Increasing the variety of perception functions for robot skin using different kinds of sensors, to allow the detection of pressure [19], temperature and sliding has become a hot topic in the field of robots. In order to allow precise measurement, the skin of robots cannot be affected by its area and structure. The combination of carbon material and a flexible substrate will be widely used in the field of robot skin in the future due to the superiority of these materials.

Here, we present a temperature sensor for robot skin, which is flexible, lightweight, easily-fabricated and low-cost. In the first section, we introduce the fabrication process of the sensors. Specifically, the sensor is made up of four layers: the insulating layer, sensitive layer, conductive silver wires and flexible PET. Conductive silver wires are printed to measure resistance change. The sensitive layer, which is sandwiched between the insulating layer and PET, consists simply of carbon materials. We made three temperature sensors from three popular carbon materials—reduced graphene oxide (r-GO), single-walled carbon nanotubes (SWCNTs) and multi-wall carbon nanotubes (MWCNTs)—as the sensitive materials, respectively. These three temperature sensors were compared in terms of their linearity, sensitivity and repeatability, and the sensitive layer made with r-GO showed a balanced performance. Meanwhile, the resistance of the r-GO temperature sensor barely changed under full pressure, point pressure or strip pressure. Furthermore, the r-GO temperature sensor exhibited a stable performance under different levels of deformation. Because of the insulating layer on the sensitive layer, the r-GO temperature sensor is not affected by humidity or other kinds of gases.

## 2. Fabrication of the Temperature Sensor

The temperature sensors were fabricated on a mechanical flexible PET (Nanyang Industrial Development Co., Ltd., Nanyang, China) substrate with three different carbon materials: r-GO, SWCNTs and MWCNTs (all purchased from Chinese Academy of Sciences Chengdu Organic Chemistry Co., Ltd., Chengdu, China).

**Figure 1:**
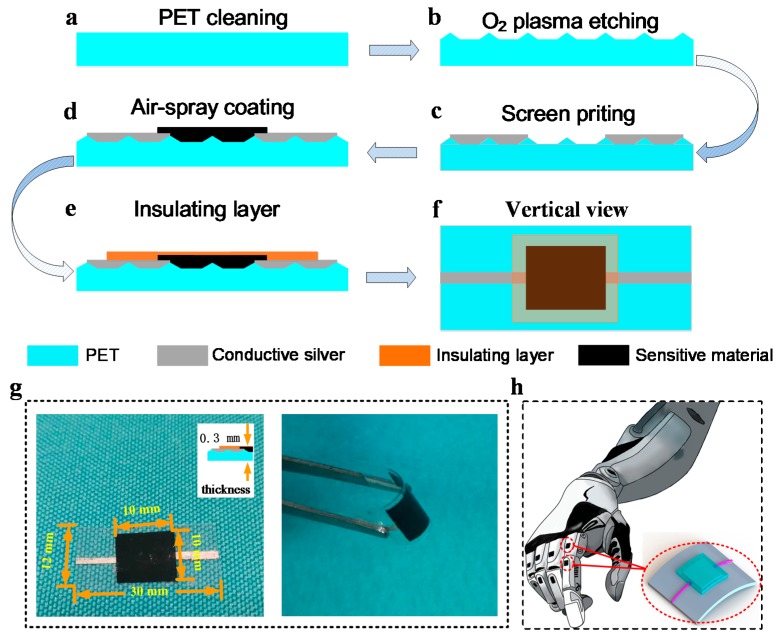
Fabrication process of the temperature sensors: (**a**) polyethylene terephthalate (PET) cleaning with acetone, alcohol and deionized (DI) water; (**b**) O_2_ plasma etching; (**c**) printing two conductive thin wires; (**d**) the fabrication of sensitive layer using air-spray coating; (**e**) the fabrication process of insulating layer; (**f**) the vertical view of the temperature sensor; (**g**) the diagram on the left is a dimensional view of the sensor, including the size of the sensitive area, the overall size and the thickness. The diagram on the left shows the good flexibility of the sensor; (**h**) One of the possible applications of the temperature sensor.

The process of the fabrication was identical, apart from the types of material used. Figure 1 illustrates the detailed process of the fabrication. Firstly, the PET was cleaned (Figure 1a) with acetone, alcohol and deionized water (DI) for five minutes for each step. In order to enhance the adhesion ability of the carbon materials to the surface of the PET, we built irregular microstructures on the surface of the PET by O_2_ plasma etching (Figure 1b) for 1 min at 280 W (Jone Wave 10). Then we used screen printing technology to print two conductive thin wires using low temperature conductive silver paste (Guangzhou Ute New Materials Co., Ltd., Guangzhou, China) on the surface of PET and heated them for half an hour at 100 °C (Figure 1c). Subsequently, the carbon material solution was coated on the microstructured PET at 110 °C to obtain thin films of carbon material by air-spray coating (Figure 1e). To control the size and thickness of the film, we covered three identical mask plates on the PET, and the spraying time was 10 s. Finally, commercial high-temperature transparent tape was pressed directly with a force of 500 N above the sensitive area as an insulating layer (Figure 1e). Figure 1f shows the vertical view of the temperature sensors. As seen in the Figure 1g, the temperature sensor showed excellent flexibility, which is important for robot skin and other objects of IoT. Figure 1h is one of the possible applications of the temperature sensor.

## 3. Results and Discussion

**Figure 2:**
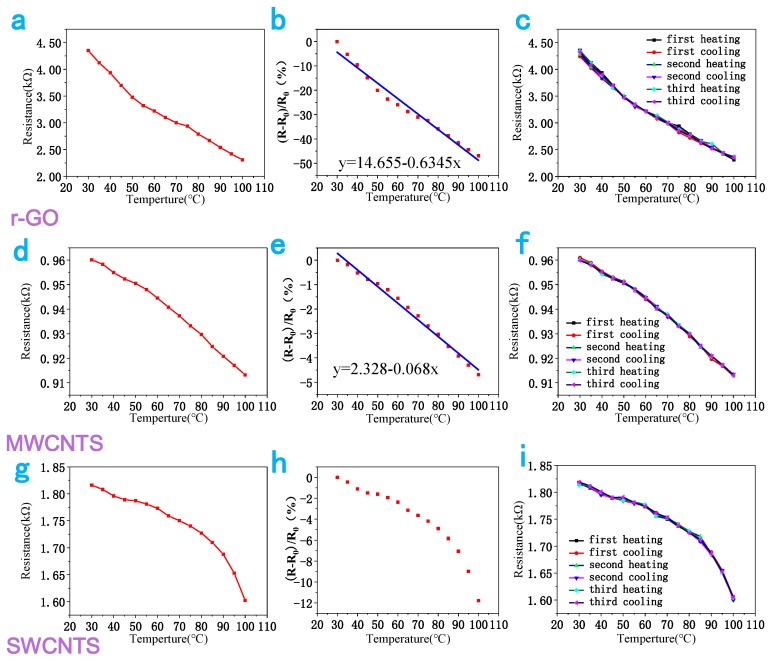
Experimental characterizations of the flexible temperature sensors: (**a**,**d**,**g**) the resistance change for r-GO, MWCNTS and SWCNTS respectively; (**b**,**e**,**h**) the relative resistance change in the three temperature sensors for temperatures from 30 °C to 100 °C; (**c**,**f**,**i**) the resistance responses of these three sensors to three cycles of heating and cooling.

To test the performance of the three temperature sensors, the resistance change was measured using a digital multimeter (UNI-T UT61) during a change in temperature from 30 °C to 100 °C. The resistance change was recorded with each measurement step of 5 °C, and the temperature was maintained at each step of 5 degrees Celsius for 5 min to observe the change in resistance. Figure 2 shows the representative experimental characterizations of the temperature sensors, whose sensitive material is the r-GO, MWCNTS and SWCNTS, respectively. Figure 2a,d,g illustrates the resistance change with the temperature for the three different temperature sensors. It clearly indicates that the curves of the graphene and MWCNTS temperature sensors are closer to linear, which is more suitable for a temperature sensor. The temperature coefficient of the resistance (TCR) is often used to describe the temperature-sensitive properties popularly known as sensitivity [20]:

*TCR* = (*R* − *R*_0_)/(*R*_0_·Δ*T*), (1)

where *R*, *R*_0_ and Δ*T* are the measured resistance, the resistance at 30 °C, and the deviation in temperature (°C) from 30 °C, respectively [21]. The change in resistance with temperature is illustrated in Figure 2b,e,h. As can be seen from these figures, the variation in resistance with temperature of the r-GO and MWCNTS decreases linearly, while the curve of SWCNTS sensor is nonlinear. In addition, the extracted corresponding sensitivities were found to be 0.6345% and 0.068% per centigrade for the r-GO and MWCNTS sensors, respectively, using linear curve fitting. Furthermore, the temperature sensors showed good repeatability and stability, as illustrated in Figure 2c,f,i. After three cycles of heating and cooling, the curve of the resistance change with temperature was almost unchanged, which is important for application of the temperature sensors.

Based on the above factors, we suggest that r-GO is more suitable for the sensitive material of the temperature sensor. So, all of the subsequent analyses are based on the r-GO temperature sensor.

The change in resistance of r-GO with temperature has been mentioned in many articles. The temperature resistance effect of graphene has received considerable attention because of its unique electrical properties. Graphene is a magical material and its magic lies in its semi-metal make-up with a zero-band gap. Interestingly, in some literature, with the change of temperature, the resistance change of graphene was shown to be in the opposite direction [15,22]. The reason for this phenomenon is due to the semiconductor and metal properties of graphene. When graphene exhibits semiconducting properties, the dependence of the resistance on the temperature is determined by its thermally activated charge carriers. As the temperature increases, the mobility of the charge carriers increases, and thus, the resistance decreases. When graphene exhibits metal properties, the dependence of the resistance on temperature is determined by charge carrier scattering. As the temperature increases, the probability of carrier scattering is increased, which causes a decrease in mobility of the charge carriers and an increase in resistance. The metal properties often appear in graphene produced by the physical method, while the semiconducting properties usually occurred in graphene produced by the chemical method [23]. Chemical method is difficult to reduce graphene oxide completely, and some oxygen and hydroxyl groups will stay on the surface of the r-GO, which will lead the change from zero-gap graphene to finite-gap semiconductor [24]. The r-GO used in this paper was made by chemical method, and the performance of our r-GO temperature sensor is consistent with the above theory, which shows semiconducting properties. The relationship between the resistance and temperature of r-GO can be expressed by the following equation [25]:

*R* = *R*_1_*e^E^_a_*^/2*KT*^, (2)

where *R*, *T* and *R*_1_ represent the measured resistance, the ambient temperature of the sensor and the resistance at an infinite temperature. *K* and *E_a_* stand for the Boltzmann constant and the thermal activation energy, respectively. A negative temperature coefficient of resistance can be summarized by this equation, and it is consistent with the measurement result.

**Figure 3:**
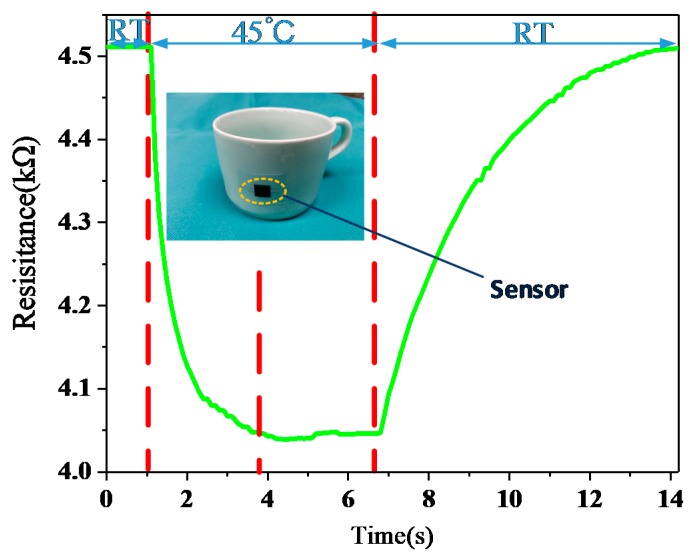
Response and recovery curve of the temperature sensor between room temperature (RT) and 45 °C. The inset is the cup with the attached sensor.

Figure 3 shows the response time (RES) and recovery time (REC) of the temperature sensor. When the temperature sensor was put in a room temperature (RT) environment, the resistance of the sensor remained at 4.511 kΩ. After a period of time, the sensor was attached to a cup containing 45 °C water, and the resistance of the sensor decreased rapidly and stayed at around 4.040 kΩ—this is a drop of about 1.2 s. This means the response time (RES) was about 1.2 s. After being kept on the heating table for a while, the temperature sensor was removed to room temperature and the sensor resistance returned to 4.511 kΩ.

The visualized parameters of the r-GO temperature sensors that we designed as well as other flexible temperature sensors are shown in Table 1. From this table, we conclude that the r-GO temperature sensor that we made has a higher sensitivity compared to other sensors and it can be tested at temperatures up to 100 °C.

The temperature sensor must have good mechanical properties so that it can be distributed on any surface of the measured objects with negligible resistance changes. This problem has been taken into account in the fabrication of the sensor. In order to minimize the influence of pressure on the resistance of r-GO, we used a very strong force to press the sensitive area in the process of making the insulating layer, to reduce the distance between the graphene layers.

**Figure 4:**
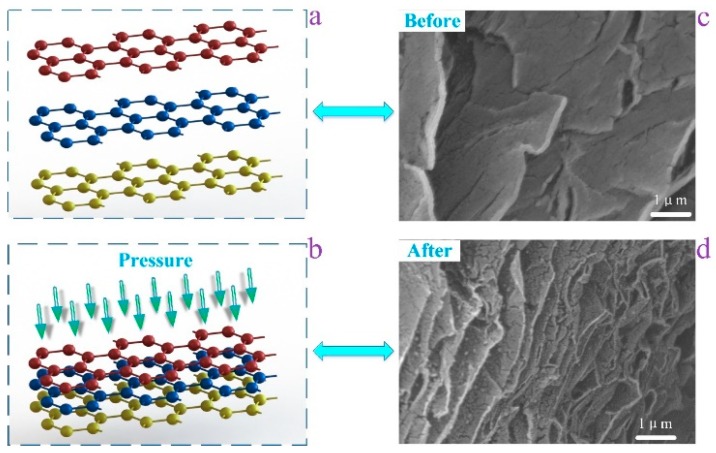
Layered structure model and SEM of r-GO: (**a**) schematic showing the layered structure model before pressure; (**b**) schematic illustrating the layered structure after pressure; (**c**) SEM image of the r-GO before pressure; (**d**) SEM image of the r-GO after pressure.

**Figure 5:**
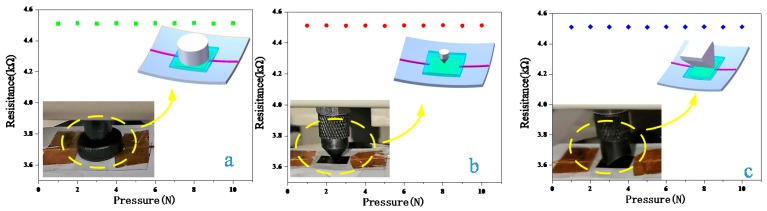
The relationship between the resistance of the temperature sensor and the applied pressure. The inset is a field photograph of the pressure test and a schematic diagram of the application of pressure on the sensitive area: (**a**) the resistance change when applying pressure to the whole sensitive area of the sensor, from 1 N to 10 N; (**b**) the resistance change when applying pressure only on a point, from 1 N to 10 N; (**c**) the resistance change when applying pressure on a strip area by a wedge, from 1 N to 10 N.

In this case, the pressure generated by the smaller contact force did not change the distance between the r-GO layers and the resistance of the r-GO was only be slightly affected. Figure 4 shows the model of this process. From the schematic, we can visualize the change in the distance between the graphene layers before and after the application of pressure. Figure 4c,d illustrate the SEM image of the r-GO. The r-GO layer before applying pressure is relatively loose, and the layers become tight after applying pressure. Furthermore, to verify whether that pressure has an effect on the r-GO sensor, the resistance change of this sensor was measured under different kinds of pressure, from 1 N to 10 N, including the pressure over the entire sensitive area, the pressure on a single point and the pressure on a strip area. Different object shapes that exert pressure on the sensor were used to simulate the type of pressure. Figure 5a shows the full pressure on the sensor, and the object applying pressure is circular and bigger than the sensitive area. Figure 5b indicates the application of point pressure on the temperature sensor, from 1 N to 10 N. We also made use of a wedge to apply a strip pressure to observe the resistance change; the simulated diagram is in Figure 5c. The resistance of the temperature sensor was almost unchanged when we applied different kinds of external forces, showing that the sensor performs well under small pressures.

**Figure 6:**
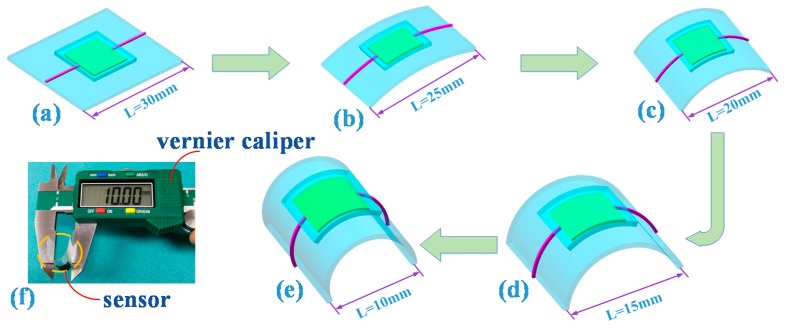
The length of the long side of the sensor (L) and bending schematic diagram of the temperature sensor: (**a**) L = 30 mm; (**b**) L = 25 mm; (**c**) L = 20 mm; (**d**) L = 15 mm; (**e**) L = 10 mm; (**f**) the way to control the bending.

**Figure 7:**
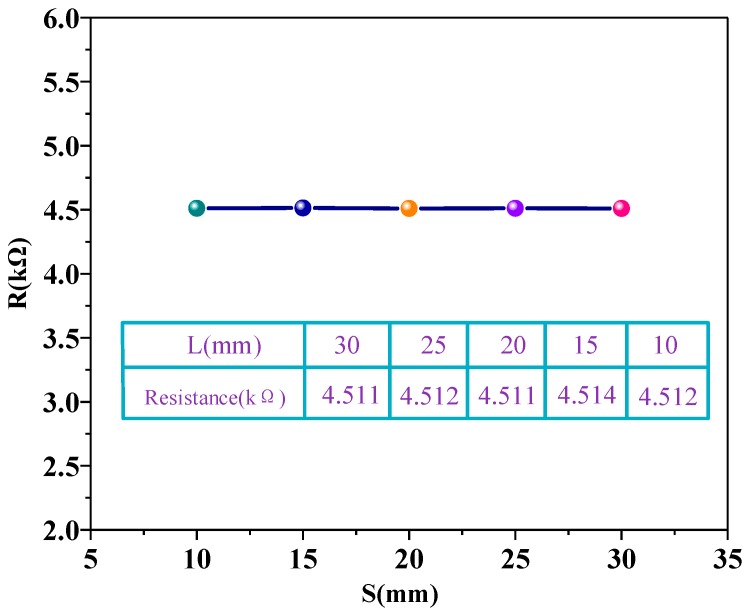
The resistance change in the r-GO sensor when bending. The table in this figure shows the specific change in resistance.

To test whether the temperature sensors were influenced by deformation, we measured the resistance change when the sensor was bent at different angles. The bending angle was represented by the length of the long side of the sensor (L). A Vernier caliper was used to control the length of L. Figure 6 illustrates the length of the long side of the sensor (L). The bending angle is inversely proportional to the L. The total length of the sensor is 30 mm, and the sensor was bent from 30 mm to 10 mm to observe the resistance change. The concrete schematic diagrams of bending are illustrated in Figure 6. As shown in Figure 7, the resistance of the temperature sensor was almost unchanged under different bending loads, which indicates that bending has little influence on the temperature sensor, and thus, it can be ignored. During the bending process, the graphene film may generate a tunneling effect, which cancels out the deformation caused by the partial bending and the scalability of the carbon–carbon bond spacing, so that the graphene has little change in the conductive property during bending [31].

The temperature sensor was almost unaffected by surrounding environments like humidity or other gases. Because of the insulating layer on the sensitive area, the r-GO does not absorb water vapor or other gases in the environment. When we put the sensor in gases such as O_2_, CO_2_, NH_3_, vapors of ethanol, formaldehyde, methanol, acetone, or humidity, its resistance did not change.

## 4. Conclusions

Three kinds of temperature sensors were demonstrated in this study, with sensitive materials made of r-GO, MWCNTS and SWCNTS. In order to determine the most suitable material to make a temperature sensor, the linearity, sensitivity and repeatability of the three materials were compared. The results showed that the r-GO temperature sensor had the most balanced performance, exhibiting a better linearity and high sensitivity of approximately 0.6345% per centigrade, and remaining stable after three cycles of heating and cooling. Furthermore, the r-GO temperature sensor showed good mechanical properties and could be bent in different angles with negligible resistance change, which means it can be distributed on any surface of measured objects. The ability to bend is also important for applications using temperature sensors in practice. Also, the r-GO temperature sensor almost unaffected by surrounding environments, like humidity or other gases. The temperature sensor we presented is flexible, lightweight, easily-fabricated and low-cost, moreover, different external pressure stimuli do not influence its performance. All the above factors show that the r-GO temperature sensor is suitable for robot skins and electronic skins and it can be widely used in the IoT. However, the RES of the temperature sensor is not so good. Moreover, only slight pressure has no influence on the performance of the r-GO sensor. The resistance of the sensor will be affected when the external pressure is too high. To deal with this problem, we will try and design new structures and take advantage of other temperature-sensitive materials, such as metal-modified graphene or a mixture of graphene and polymers in the future.

## Figures and Tables

**Table 1:** Some kinds of flexible temperature sensors and their performances.

Sensitive Material	Range of Measurement (°C)	Linearity	Sensitivity	Response Time	Ref.
reduced graphene oxide (r-GO)	30–100	Yes	0.6345% °C^−1^	1.2 s	ours
r-GO filled cellulose films	25–80	Yes	/	/	[26]
Carbon nanotube	21–80	Yes	0.25% °C^−1^	1~2 s	[27]
single-walled carbon nanotubes (SWCNT)	0–80	Yes	/	/	[28]
Ag	20–60	Yes	0.223% °C^−1^	<80 ms	[29]
Ni fibers	0–100	Yes	0.48% °C^−1^	/	[30]
Au	30–80	Yes	0.15 °C^−1^	1.7–2.3 s	[13]
